# Address

**Published:** 1879-07

**Authors:** F. Peabody


### THE DENTAL REGISTER.

VOL. XXXIII.] JULY, 1879. No 7.

### ADDRESS.

BY DR. F. PEABODY, PRESIDENT.

*Read before the Kentucky State Dental Association.*

*Gentlemen of the Kentucky State Dental Association:*—Under the blessing of Divine Providence, we are permitted to assemble again in this, our annual reunion; and our hearts should be filled with gratitude, for the privilege thus extended. We have since our last meeting passed through a year of peace and plenty. Death has not diminished our numbers, and, while we are one year further on in life, we are one year advanced in knowledge and experience. A hearty welcome and cordial greeting are extended to one and all of the members of this association, and we trust that our stay together, short though it be, may be both pleasant and profitable. There is no period during the year of our professional labors that is more pleasant than this annual gathering. We meet for general improvement. For the time, cares and anxieties are laid aside. Here we give ourselves freely to fraternal and professional intercourse, anxious to receive all that may be imparted of good, while we enter into a kindly and generous discussion of whatever may be brought before us, earnestly seeking to improve ourselves and elevate our calling. It is much to be regretted that of those practicing dentistry in this state a greater number are not interested in this association. Associated efforts are productive of more good than those of any other character, and stupid, indeed must be the meeting or dull the member who, when he returns to his home, does not feel that he carries with him something of value, something that will enable him to pass more smoothly over the shoals of the future, and in some degree to avoid the quicksands and rocks he may have encountered heretofore. We have many good men in this state, who, were they interested in these meetings, could assist us much in our deliberations. Out of something like three hundred members of the profession in Kentucky, in this, our ninth annual meeting, this association has a membership of less than twenty-five. It does not redound to the credi t of the state that so few are interested in advancement and improvement. Most of the states north, east and-west of us can make a better report of the condition of their associations than we are able to do, and it is time we were awakening to the importance of strengthening ourselves; and in view of these facts I would recommend that steps of a proper character be taken to arouse from their apathy the good and true men within our commonwealth, and induce them to aid us with their presence and counsel in bringing our association on a par at least with our sister states.

Our pathway through a conscientious professional life is by no means smooth or easy. We are constantly encountering perplexing peculiarities, and are as constantly harassed by doubts as to the proper course to pursue, and thus we need the aid of those of large experience and enlightened views to tide us over our troubles; and it is here, in these associations, that we meet them; here that we exchange views and suggestions; here that we gain ideas that will open new fields to us, and enable us to do more of good to our fellow-creatures. We attempt now what practitioners of thirty years ago did not dream of. We save where they lost. We make efforts for the preservation of the natural organs, where they replaced by artificial. All our literature of to-day tends toward the conservative course. We fail frequently, yet we accomplish much; but we must not rest satisfied with this. We must go farther, and, if possible, look more closely into the causes of disease, and make better efforts toward prevention rather than restoration. Something has been done in this direction, and that encourages us to hope more may be accomplished by diligent efforts in the right spirit. It is often remarked that our profession is in its infancy. In years it counts but few, but in strength it is a Hercules, and the power it has shown, the strides it has made during the past few years (sometimes backward, it is true, to correct its false steps, yet ever onward) are something remarkable even in this progressive age. Nowadays nothing is permitted to stand still; everything advances or dies; retrogression is death, and we may justly congratulate ourselves that we have not been crab-like in our movements. and that dissolution is not at hand. So much has been accomplished of late years, it would not be reasonable to expect that we could keep up the same rapidity of advance. We must, for a while, go slowly, that we may thoroughly digest that which we have on hand and see that our advance is of a healthy character, as all that is truly good must withstand the most thorough and rigid investigation.

From many of our associates here we expect to learn much that is valuable, and there are none who may not contribute something to the general fund of information. Our calling is in many respects manipulative in its character; and those who have the most wisdom and the greatest experience may learn from others, who do not care to come before the association in discussion, points and practices that will be of advantage. No one mind is capable of grasping everything. No one hand capable of executing perfectly all that it undertakes. We come together to learn; here we speak freely and fraternally; nothing is kept back; each adds his mite, and all are benefited.

In former days each item of advantage or attainment that enabled one to do more or better than his neighbor was zealously guarded from the eyes of others. No spirit of liberality or generosity existed; colleges were not in existence; associations were unknown; information was imparted only at a price; selfishness and secrecy were the rule. Those were the days of darkness and bigotry; the days of a trade, not a profession. Now all is changed. Our associations have disseminated knowledge to all. The true spirit of culture and courtesy, improvement and advancement animates all who are truly professional in mind and character. We join in an eager and earnest desire to add to our store of knowledge that our sphere of usefulness may be more expanded, and that we may more surely approach that degree of excellence and attainment that we fully believe exists in the horizon of a golden future. From the programme that has been prepared we may reasonably expect to have a thorough ventilation of subjects most interesting to us. We trust all of the various committees have come with papers on the subjects to which they are appointed, and it is earnestly hoped that all the members of the association and those in whom the courtesies of the floor may be extended will aid by their contributions and assist in promoting the harmony and good-fellowship of this meeting. For the twelve months passed, as far as it is known, fraternal feeling and conformity to the laws have been the rule. Nothing within our own membership in the way of improvement has been presented that is particularly worthy of mention. Many good articles have appeared in the pages of the Cosmos, and particular attention is called to papers from the pens of Dr. Frank Abbott, on caries of human teeth; Dr. C. F. W. Bodecker, on the distribution of living matter in human dentine, cement and enamel; and Dr. J. W. Farrar, on aveolar abscess and necrosis, sulphuric acid versus creosote. These papers have been prepared with care, evincing much research and study, and will repay any who will peruse them thoroughly. No new applications or suggestions have been brought forth since our last meeting which are of much importance. Boracic acid is spoken of in place of permanganate of potassæ, acting in the same manner, leaving no stain. As a therapeutic agent it may possess advantages. But little light has been thrown on the disease termed pyorrhœa alveolaris, which term is exceedingly vague and by no means satisfactory. We are groping much in the dark in regard to this trouble and its successful treatment, and until the profession make a marked distinction between the disease proper and the effects of the encroachments of the salivary calculus, but little of a definite character can be expected. The subject of the "new departure" has, during the past year, attracted much attention. Many able papers, both pro. and con. have been presented to the profession. Many of the propositions are set forth by the advocates of the creed, and are hard to accept, particularly the electro chemical theory. Much injury has been done by the broad acceptance of the views of the originators of this subject. The Scientific American, a paper largely circulated and read, by quoting some of the views of the extremists only, has led many to believe that gold must no longer be used as a filling under any circumstances. But while in this direction much that is evil has resulted, much good has also been accomplished by the new views. Many have been led to investigate the subject more closely than heretofore as to the value of various articles proposed as filling materials, and more care has been bestowed on the compounding of plastic materials for fillings. Aside from this there has been a much greater tendency to return to the use of soft or non-cohesive foil by those who had an exclusive leaning toward cohesive. When fillings of soft foil, made by the older members of the profession, twenty or thirty years ago, without the advantages of the present day, are pointed to as monuments of its value and its saving power, it leads the reflecting mind to think such an article can not be the poorest to use in producing desired results. Unfortunately many members of our profession are inclined to run to extremes, and to advocate with avidity almost anything that an acknowledged leader may pronounce as valuable. Thus grave mistakes are made. If there is any profession in which the eclectic principle should preponderate, ours is surely that one; the cases we meet are so variable, the constitutional tendencies so extreme, a good judgment is absolutely necessary to accomplish the most good. Where all materials have their value, the use of one to the exclusion of others, regardless of surrounding circumstances, can but be productive of unpleasant sequences. No one should be an extremist in anything. Broad liberality in all views, and charity for the shortcomings of others, with calm, unbiased judgment, will do much to keep us from error. The efforts at improvements in plastic materials have been marked. The porcelain cement of Fletcher works well, and promises to be an advance on other materials of a similar character. Much is claimed for it, however, that it will not justify. It is of too recent introduction to know much of its permanency. In the improvement in amalgams, while something has been accomplished in regard to color, it is questionable whether in saving qualities any of them are better than those used before the efforts at improvement were attempted; still the general advance toward the ideal filling is encouraging. The law which was passed at the last meeting of the Legislature, to regulate the practice of dentistry, must ultimately be productive of good. It would have been better had it been passed some years sooner. It has been felt that something must be done to prevent impostors and charlatans from imposing on the public to the detriment of the standing of the profession. In many of the states protective laws have been passed, and, as the necessity of this is seen year by year, other states are pursuing a similar course. Indiana, at the session of its General Assembly this year, passed a dental law, but no information has been conveyed to this association as to its provisions. England has passed a dental registration law, making it incumbent on all practicing dentistry to be registered; the application must be accompanied by qualification, with date, medical authority and by whom granted; date of commencement of practice, county and counties wherein practice has been carried on, and evidence of good character; and these applicants can not be registered until their certificates have been approved or recognized by the general medical council. Up to the present no applications have been made to the state examiners for license under the law to practice in this state, and no prosecutions for infringement of the law have been reported. Since the commencement of this year one of the brightest ornaments of our profession has passed away, and we are called upon to mourn the loss of one whom all revered, all honored. A gentleman of large information, a ripe scholar, a close investigator and thorough student, for years a teacher in one of our foremost institutions of education, he has done much to instruct those entering the profession, ever a prominent member of the associations with which he was connected, a ready debator and instructive writer, interested in all that pertained to his profession, earnestly seeking after the truth, closely investigating the more intricate hidden mysteries that environ it, his aim being to instruct himself and others and elevate the profession to a higher standard; Professor McQuillan did not shrink from any duty that devolved upon him. No one labored more assiduously; his whole life was given to investigation and instruction, thinking less of himself than of his life work. And while he can be with and of us no more, his name and work will stand as a monument of emulation to those who have a true professional life in view.

A word on education, and this paper, already too long, will close. The American dentist stands conspicuously in advance of the European. This is almost entirely owing to the superiority of his operations in gold. In knowledge, as acquired in colleges, it is doubtful if the educated European is not his equal, perhaps his superior. The English have lately shown a lively interest in pushing the profession in their country to the front. They are receiving all the advantages we have heretofore possessed in operating, and their collegiate course is much more thorough than in America. Few, if any, of the graduates of our colleges could answer the questions given to the English candidate for examination. If the standard of education is not raised here we will soon have to look to our laurels. One great advantage they possess is the requirement of a good general education before an applicant can be admitted to a course of lectures. Young men are not there taken from the plow and workshop, with merely the rudiments of an English education, and forced through college in one or two years to be turned out doctors or dentists. Here one may enter the laboratory, and, after spending a few months learning the mechanical art only, nothing prevents him hanging out his sign to impose on a public not capable of discriminating between a skillful operation and a bungling one. The laws that have passed in the various states will, in a measure, correct this where these laws exist, and a candidate for dental practice will find hereafter that he must educate himself to the required standard in order to practice in these States, but in many states no law exists, and it is not enough that we merely seek to protect our own community. The benefit derived from proper qualifications should be felt all over the land. When we have received our degree at the college, we have simply learned how to study, and our life as a student has then but fairly commenced. Too many are apt to think at that point their education is finished. We wish to keep no one from entering our ranks. We extend the right hand of fellowship to all who have the interest of our profession at heart, but we do wish to feel that the densist of the future will not only honor his profession, but the profession will be held in such esteem that it will honor him.

